# New Jersey State Dental Society

**Published:** 1888-12

**Authors:** 


### Reports of Society Meetings.

### NEW JERSEY STATE DENTAL SOCIETY.

JULY, 1888.

ESPECIALLY REPORTED FOR THE INDEPENDENT PRACTITIONER.

### DISCUSSION ON DR. B. HOLLY SMITH'S PAPER ON "PROFESSIONAL PATENTS."

*Dr. Ivory*—I especially like the last part of the paper, showing what wonderful things inventors have done for this universe. Any person who makes an invention must give his time and his money to it, and should be paid for his invention in order to get back what he has spent upon it. A dentist is protected in his work more than is the man who takes his patent in the Patent Office at Washington, and to consider it unprofessional conduct for a dentist to try to get back his outlay on an invention is wrong. Here in America we are protected as no other nation is protected. And it is by this protection that we thrive. S. S. White was a man that we should be proud of. If it were not for him you would not have the beautiful instruments that you have to-day to do your work, and when you buy these of the firm he established you get your full money value. He was at one time a dentist, and in this other branch of his calling he saw a profit for himself and his professional brothers as well, and he went into it and made a success of it.

I have just noticed a statement in one of the papers that J. H. Lippincott, of Pittsburgh and New York, has paid about a million dollars for a patent. Edison has sold one invention for a million of dollars. No doubt if Dr. Smith could patent an invention and get a million of dollars for it, he would accept the money with thanks.

*Dr. Atkinson*—Whenever this question comes up, there is such a manifest dearth of evidence that either side have grasped fully the foundation principles involved, that it is almost useless to attempt to speak to the benefit of listeners. The great difficulty is that they do not distinguish between law and legislation, between the eternal varieties and the contracts which legislators make to govern the commercial aspect of our interests. The whole patent system is robbery from the beginning to the end; and every inventor, with the exception of a few, has been robbed by the commercial spirit of the men who get control of the invention. Are we men enough to be fair? is the question.

The very fact that we have to eat something that has just died is a serious question. The bread and butter question is horrible to contemplate. And the bread and butter question being based upon financial advantage, who is entitled to the advantage? When we graduate a man, he is entitled to all the rights, privileges and immunities belonging to the grade which he has attained. Iniquity is at the bottom of the whole of it? What right have we to any rights that are not right at all?

*Dr. Hayhurst*—Perhaps, looking a little beyond the narrow bounds of the profession, which embraces only a small proportion of mankind, the mention of the greatest evil in the world to-day is contained in the expression of the big fish eating up the little fish. It is not only in the profession, but in the world at large everywhere, this great eternal grasping for something more than you have got. It is exemplified in the great monopolies of our country, in the railroad system, in the telegraph system and in the trusts, combining to appropriate everything that is good. And this patent system has been reduced to the level. As has been observed it is very seldom that the man whose brain was fertile enough to make an invention received the benefit of it; but it goes into the maw of these trusts or monopolies which absorb and take all the benefit, and the poor inventor goes penniless and supperless. It is that evil which we have to strike at in the profession, and it was that which this paper endeavored to depict; and so far as its general course is concerned I think it was an admirable paper. In its general trend it is just what we need. If all the human family had that which belonged to them, that which would be beneficial to them, it would be all right; but when the rights of the human family are taken and sold as merchandise and kept away from the masses and any benefit derived from them has to be paid for, it is an evil. You had that evil in the Goodyear patent. I received a circular not long ago with the very same mark as in that case, and signed by the very same man, threatening to prosecute me for putting on Richmond crowns. This is an evil, and it grows out of the patent system. I say that it is the bounden duty of every true man within the bounds of society to set his face like a flint against the extension, the support and the maintenance of this patent system.

*Dr. Thayer*—The profession of dentistry would not be to-day what it is if it had not been for the inventive genuis of some gentleman in the ranks. You know that is true. Eighteen years ago there came into my possession an instrument that has done more good in this profession than any one instrument that has ever been invented, viz.: the dental engine. If you perform an operation in dentistry you are entitled to a fair compensation for it, and if you invent some new method of making an operation which costs you time and money, you should protect yourself by taking out a patent. If it is right for a man to earn an honest penny in one way, it is right for him to earn an honest penny in another way.

I think I can speak on this subject without any prejudice, because I never have invented anything. I have not ability to invent anything that would be of benefit to the dental profession, and I have no interest in supporting this side of the subject; but I do believe in throwing aside all this ethical nonsense and giving the man who has the ability to invent something that is valuable his just dues in connection with it. If it is a benefit to the profession they will buy it and use it for their profit; if it is of no value they can let it alone. There are a thousand ways in which inventive genuis can circumvent it just as Dr. Parr has circumvented the patents in his tooth crown, and as Dr. Brown has done the same in his porcelain crown and bridge work.

*Dr. Smith*—I will say that I agree with the gentleman who spoke last, that dentistry would not be what it is to-day without the inventive genius which has aided it so wonderfully, and I refer in the paper to the benefits received from such inventions. I want to ask you if in surgery or in medicine, where there can be no opportunity for such regard as it is claimed is necessary to stimulate inventive genius, is there any dearth of inventions or inventors? What did Flint get for his splint? What did the inventor of the laryngoscope get for his invention? They got credit before the medical profession and before the world at large for having done something, and a great deal, to relieve suffering humanity, and that I claim is a reward. When you say throw ethics to the dogs, you rob us of that which we hold dear to our hearts, and you would rob the dental profession of that which makes it a profession, and reduce its members to the level of trademen.

The subject was here passed.

DISCUSSION OF DR. THAYER'S PAPER.

*Dr. Genese*—Mr. President and gentlemen, we have, as Dr. Thayer has said, many sceptics in regard to the saving of exposed pulps, and I can only say that I have a record of over two hundred cases of pulp capping within the last ten years, and I was able to say, in a paper read before the New York Odontological Society a short time ago, that some of these teeth, the nerves of which had been capped during the last six years, were to my knowledge still doing good service. The avoidance of pressure in the capping of nerves is of the greatest importance. I do not use any metallic substance in capping nerves ; I use a preparation that will, while soft, take the form of the nerve tissue when it is hardening. One of the preparations that Dr. Thayer has mentioned I have had considerable experience with; that is the sulphate of lime. I have very rarely found that to fail in hastening the process of suppuration. I have discarded acon te altogether, for the reason that we get so great an effect from small doses in some patients. In one case I used a very small portion of the diluted tincture of aconite, and the next day I was visited and accused of poisoning the gentleman. I had placed in the cavity a very small portion of aconite on a bit of cotton, yet the effect was so great in a short time that I never could persuade the gentleman that he had not been poisoned.

The cases of sixth year molar exposure of the nerve have been the greatest successes that I have had in capping. The most failures have taken place in cases where the nerve was excised. The principal thing that I have used in combination with capping has been the oxide of zinc mixed with carbonate of lime and cocaine. I will show you that exposed pulps capped with this preparation will be perfectly oblivious to the action of cold or warmth. I think the exclusion of air and pressure in treating exposed nerves before we attempt to fill will preserve a very large number that would be otherwise lost.

*Dr. Meeker*—I consider myself densely ignorant in regard to homœopathic therapeutics, but I must say that Dr. Thayer's exposition of the homœopathic law of cure has been the best that I have heard.

I have used nearly all the remedies mentioned by Dr. Thayer.

*Dr. Ivory*—In every case of exposure of a nerve there is a consequent inflammation from various causes, such as the fluids of the mouth coming in contact with the susceptible and sensitive nerve tissue, and the operation of filling a tooth immediately on finding a nerve uncovered is, I think, altogether unscientific and unprofessional; for the reason that when you put in a filling you hermetically seal the cavity with your gold, cement or amalgam, and there is no way for the inflammatory products to escape. But by the use of medicines, such as aconite or iodine or carbolic acid, and leaving it open to the influence of the air—and the air is one of the principal agents in the healing process; if you have a sore on your hand it is necessary for it to have air in order to heal properly—if you treat it in this way the inflammation may subside and the operation of filling be successful. If you put in a filling immediately you do wrong, but if you leave it so that the air can come in contact with the tissue for a few days, and then put in your filling, you will have better results than if you fill on the moment.

*Dr. James Truman*—Mr. Chairman, probably I know as little as any other person present in regard to the action of homœopathic medicines. I give homœopathy credit for many good things, one of which is the liberalizing of the old school of medicine, not only in their treatment, but in their use of remedies; but I do not understand the principle of homœopathy, and I have never been able to understand it so that I could bring it into practice satisfactorily to myself. I also do not understand one assertion which I think the essayist made, that aconite increases the rapidity of the circulation, and that he could remove inflammation or pulpitis by its use, through overcoming the static condition of the blood which necessarily precedes positive inflammation. My idea of the action of aconite is that it is a cardiac depressor; that it acts probably through sympathy with the vaso-motor nerves, and, while it produces irritation of the vessels, will necessarily slow the action of the blood in those vessels. Therefore I do not understand his view of the matter. He may be correct, but it is not so generally understood. I do not comprehend his assertion that he can save nine-tenths of the exposed pulps by that sort of treatment. My own view of the matter is—as he probably will agree, because he said that freshly exposed pulps could be better treated than those in which inflammation had supervened—that it is hardly possible to save an exposed pulp to the extent that has been represented by many in the profession and in this paper, except the condition of the teeth is of a certain favorable character. It seems to me unphilosophical to suppose that a pulp in a highly inflamed condition can be capped and its vitality retained. I do not think it can be done except in certain conditions, conditions of great vitality of the individual, exemplified in very dense teeth, and in those cases softening of the teeth precedes the capping. I have seen recently, under the treatment of Dr. Jack, in Phila., a few such cases; the teeth were extraordinarily dense in structure, showing great vitality in the individual. How many cases of this favorable character come into our hands? Those most frequently met with are of an entirely opposite condition, and we have to meet this condition, and not the others, which are comparatively rare.

In regard to homœopathic treatment, I am really glad that the New Jersey State Society is liberal enough to admit a paper upon this subject. I believe in broadening our views by an honest examination into these questions. Certainly homœopathy, as I understand it, is about as foreign to my conception of therapeutic treatment as anything possibly can be; but I always hold myself open to conviction.

*Dr. Luckey*—Mr. President, there is no occasion for me to rise to say anything on this subject, about which I do not pretend to know much; but I have noticed a very great diversity of opinion among the gentlemen present in regard to their methods of treatment, and in regard to the treatment of pulps generally. And I want to take this occasion to call to order two or three gentlemen who have been talking about nerves here. I am very sensitive about that term. We are dentists; and we treat not only the teeth, but dental pulps—not nerves. The public, and most of the dental profession should be made to understand that we have nothing to do with the treatment of nerves except as a part of the dental pulp.

As to the treatment of them, there seems to be great diversity of opinion, especially between Dr. Genese and Dr. Ivory. Dr. Ivory believes that air should be allowed to come in contact with the pulp. We have been taught that the air is full of microbes; and if anything will start up inflammation in a dental pulp, the admission of air carrying these microbes will do it. Dr. Genese covers his pulps with thymol cocaine, and says the teeth will not respond to thermal changes. I believe that is true. But the question is, How does the pulp respond the next day? We know that cocaine will quiet the sensitiveness of soft tissue, but the result is not permanent; and that, unless some non-conducting substance is interposed between the pulp and the outer air, there will be a response. In treatment of inflamed pulps, Dr. Thayer has tried to lead us to believe that it is possible to save nine out of every ten when they are in a highly inflamed condition. Is that your statement?

*Dr. Thayer*—That is the statement.

*Dr. Luckey*—I do not believe it is possible to save two out of ten highly inflamed pulps. Congestion of the pulp causes such an engorgement of those minute capillary vessels as to cause constriction of the pulp at the foramen, and unless the foramen is very large death of the pulp is sure to ensue. You may treat and cap it, and you may keep it quiet, but it does not follow that it is alive. There are cases of congestion where the pulp can be saved; but I think it is a very safe rule to destroy it. When a man says arsenious acid, nor anything of similar nature, shall be used in his office for destroying pulps, he is not only doing injustice to himself, but he is doing injustice to his patient. There are many cases coming to the hands of the practitioner where it is far better for both patient and practitioner to destroy the pulps than to try to save them.

*Dr. Thayer*—With regard to the remark of the gentleman who said that he did not believe it possible to save the large percentage of pulps that was claimed by your speaker, I think your speaker stated distinctly and positively, that it was highly injurious and very hazardous to attempt to cap a pulp while there was a vestige of pulpitis. Your speaker never caps a pulp while there is the least sign of inflammation. He reduces the inflammation and then caps, and invariably has success. I do not ask you gentlemen to believe me. I know it is a tremedous statement to make, as the subject has been understood by the profession. But I am stating what is true in my practice. I know the experience of the profession is entirely different.

*Dr. Stockton*—How do you know they are successful?

*Dr. Thayer*—By seeing my patients frequently afterwards.

*Dr. Stockton*—Do you cut into the teeth to find whether they are alive or not?

*Dr. Thayer*—No. If there has been no inflammation or pulpitis it is evident that the pulps are living.

*Dr. Genese*—The only proof of success is the examination of the cases after a considerable time has elapsed. In all our operations time alone will tell whether they are successful. Dr. Stockton asks the question, has Dr. Thayer or myself examined the capped pulps afterwards to see if they are alive. I can say that I have in very many instances. Like Dr. Thayer, I reduce the inflammation before I attempt to fill the teeth. The cap that I put on can be easily taken off a day or two afterwards and another put in its place. One day a little girl presented herself with a sixth year molar for treatment, and when I attempted to remove the disintegrated tissue the whole mass came away, leaving the pulp exposed so that I could see the pulsation. Here was a typical case to test the method that I had adopted. The child was in great pain and crying. Dr. Knapp was in my office at the time and I invited him to see the child. I showed him that even warm water —cold water was quite out of the question—could not be endured in the mouth at all. I took some of this preparation I have named on a small bit of cotton and applied it immediately over the pulp. The preparation which I use does not give the slightest pain when applied to an exposed pulp; it can be applied to a freshly cut wound without causing pain. In this case when I applied it the pain ceased. The child was allowed to rest a little while, and then water was applied warm, and the temperature of the water gradually reduced until I could apply cold water with a syringe to the pulp without giving the least pain. The effect is obtained by excluding all moisture and air and pressure from the pulp. When the patient comes again the pulp is capped and is allowed to remain in that way for several days until the capping gradually hardens, taking the form of the pulp, before any pressure of filling material is put upon it. I would say to Dr. Truman that I have found less success in teeth of very dense structure than I have with softened dentine.

*Dr. Watkins*—In answer to Dr. Stockton's question as to whether any teeth that were capped had been opened to ascertain if the pulps were dead or alive, I would say that I have opened such teeth, and instead of finding a beautiful live pulp with a nice formation of new dentine over it, I have found a nasty lot of pus in the cavity in almost every instance. They have gone on for years, and I have claimed they were grand successes, and I have told this society about the great success I have had in saving exposed pulps, no matter how badly exposed or inflamed they were; but after awhile, when the patient returned for some attention, I would open these teeth, and find a nasty putrescent pulp in almost every case. With me it is almost impossible to save an exposed pulp alive by capping. Dr. Luckey has remarked that he thought two in ten would be a very good percentage of them to save, and I heard a gentlemen whisper that he thought one in ten would be a very good percentage. I think so, too. I do not think I have saved more than one in ten.

I thought Dr. Thayer made a very good point. He said he did not kill nerves; he did something better, he tried to save them. I thought that was very good, but afterwards he went on and said he did save them. Dr. Thayer has the advantage of most of us from the fact that he treats them differently from our method, and differently from the usual method; he treats them homœopathically. It may be that he has this grand success, and that we would have the same success if we treated them as he does. I think the paper is admirably written, and I am glad it was read here.

*Dr. Genese*—I would state that the tooth I spoke of was opened within a year, and the pulp was found to be in exactly the same condition as when it was capped, and just as sensitive to cold. It has been recapped. I have seen it several times since and it is quite comfortable.

*Dr. Ottolengui*—I would like to emphasize the remarks of Dr. Luckey in regard to the use of the terms nerve and pulp. The misuse of terms in that way gives us a great deal of trouble. Dentists do not have to deal with dental nerves except as they are connected with the tissues of the tooth where there is trouble and consequent pain; and probably that would not occur were not the pulp tissue environed by the hard and unyielding substance of the tooth. I understand the pulp to be composed of connective tissue, and amenable to all the laws that govern other connective tissue, and that suppuration in it would be easily controlled except for the fact of its position.

The essayist said that when he capped these pulps he liked to have a little concave surface over it, so that there would be room for any swelling that might ensue; and afterwards he told us that he never capped a pulp until he had it cured thoroughly by treatment. Now if a tooth is thoroughly restored to health when capped, what advantage is there in leaving that space? And if it is not cured, what is the advantage of the space? If it is not cured it would have, from the irritating edges of the cavity, an additional cause of hypertrophy, and the space in the cap would be a place for hypertrophy to get into, and that would not likely to become normal tissue again.

We hear a great deal about these pulps being saved, but we hear very little about their being opened and found to be alive after a length of time; whereas we do open many pulps that have been capped by men of excellent reputation and find them dead and putrescent. I have opened some that were capped by dentists in foreign cities, men of great reputation, and my experience has been the same as Dr. Watkins' that we generally find the very worst condition of things in there; if not pus, at least an odor is noticed as soon as you open it. It is true that some teeth in which the pulps are dead never give any trouble. The pulps have been destroyed in some way, perhaps by a blow or by regulating appliances, and still the dead pulps have remained without giving trouble; but when you drill in and let that "beautiful air" in which is so nice in these cases, I think you will hear from it in a few days. I fancy that the majority of successes that we have in nerve capping are simply gained by covering up the pulp from the air and hermetically sealing it, especially in cases where there is a minute foramen. In cases where the foramen is large I do not think one in a hundred is saved alive. Therefore I think a little arsenic is handy to have around.

*Dr. Truax*—Mr. President, with all due respect to Dr. Luckey, I think it is discourteous to say to a gentleman who has been invited here to make an address or read an essay, that we do not believe the statements he makes. I have no reason to doubt Dr. Thayer's assertion, and we have no reason to doubt his reputation for truth and veracity, and while I may not believe just exactly what he does, yet I do not treat the teeth as he does, and when he comes here and makes a positive statement of results based upon entirely different methods of treatment, I think the least we can do is to accord him the courtesy of assuming that he tells the truth.

I do not think there would be any objection to that, and, with all due respect to Dr. Luckey, I think that much ought to be accorded.

*Dr. Luckey*—Perhaps I have been misunderstood. What I said was not intended to cast a reflection upon Dr. Thayer; but that I did not think it was possible to accomplish the result which he stated. Dr. Thayer, I think, will admit that he has not opened nine out of ten of the teeth he has capped for the purpose of ascertaining the condition of the pulps; therefore I think my statement will hold, that he is mistaken in his estimate of the results attained. And I think my belief in the matter will have to stand whether it is considered disrespectful to Dr. Thayer or not.

*Dr. Thayer*—Certainly, I should be very much pained indeed to open up the sacred graves of all my departed friends to ascertain if they are in fact dead. Such a demand is unwarranted, when one's patients are under constant observation, and by frequent examinations it is found that no soreness, aching, pain or pulpitis has supervened, we may know that the pulps are alive, healthy, normal, enjoying perfect physiological action. We want no resurrection or unnecessary post-mortem discussion.

*Dr. Stockton*—I have no doubt that if you did open some of the graves you would find them dead.

*Dr. Genese*—I believe that one-half of the failures in trying to save exposed pulps come from not giving them time enough before capping.

*Dr. Thayer*—I think the whole matter hinges, not upon making the operation, so much as upon the constitutional treatment before it is made. I have given in that paper the results of not less than twenty years of observation and practice.

*Dr. Wm. H. Truman*—The phrase "an exposed pulp" has always seemed to me to be a somewhat meaningless expression. Exposed pulps may be in so many different conditions that our practice and experience in treating them will necessarily vary according as we appreciate the condition. A pulp that has been recently exposed, and that has no pathological disposition, may be, I think, treated and capped with success. I have had many such cases in which I think I have been successful. But a pulp that has been exposed by the gradual approach of caries, and that is in a diseased condition, I do not think can be capped successfully by any method. There have been started certain changes in the tissue that will certainly go on, and which will, sooner or later, cause the death of the pulp. Perhaps they may be kept quiet twelve months, possibly three or four years, but death is inevitable. A careful microscopical examination of a tooth that is seriously affected by caries will show a number of changes which are constantly going on; the effort which nature makes to resist decay sometimes changes the vitality of the organ. My own experience in cases that seemed favorable in every way is that sometimes I have succeeded; but in cases where I am not sure the conditions are favorable, I do not now attempt to cap. I think it is better, when a pulp is partly dead, to apply arsenic and destroy it at once and completely. I think it is much better to do this than to put a cap over the pulp and have it die afterwards. Pulps that die under the cap leave the tooth in a worse condition than it would have been if it had been treated with arsenic in the first place.

I never like to doubt any one else's experiences. It is true that some dentists succeed by precisely the same method that others find failure in. The paper that has been read, and which I have listened to with extreme interest, has been carefully and thoughtfully written, and I do not feel that I can say much about it until I have had an opportunity to read it over. To me it is a new subject, and there are new suggestions there which have impressed me favorably, and I shall look for the publication of the paper with the extreme interest that I have listened to its reading this morning.

The paper was passed.

The next subject presented was the application of Logan Crowns by

*Dr. B. A. R. Ottolingue*—Mr. President and gentlemen: I have not prepared any paper further than what you see on the wall. (Referring to drawing illustrating the subject of tooth crowns.) I think that the subject of tooth crowns is so practical a one that I can tell you all I know about it as easily as though I were to write it beforehand.

When the Logan crown was first made, I commenced to use it, and as far as I can understand, it was simply meant to be an ordinary pivot tooth with the pivot made of metal and baked into the tooth; having the advantage of the pin being fastened in the crown, and the cement with which it was set in the root taking the place of the swelling of the old wooden pin from moisture. When I first got hold of the Logan crowns, I put them in with the idea that the oxyphosphate cement was going to hold them in place. I think the first one I set came out in about two weeks. The next one I think did a little better; but I believe that all the crowns which I set with oxyphosphate cement came out in course of time. Then I tried amalgam; and the last one set with amalgam came out last week. I then began putting them in with gutta-percha; and most of those I set with gutta-percha have kept their places. Still I was not satisfied that I was doing more than haphazard work, and I wanted to do something better. But before coming to what I consider was an improvement upon the old method, I want to show you how the old method was wrong. I refer to those put in with oxyphosphate. The pin was pointed; and the canal, as represented in this chart, is generally a perfectly cylindrical hole. What is the result? The only point at which this pin touches the walls of the canal, if it touches them at all, is the point where it first enters the root. Consequently, the deeper you cut down the canal, the wider becomes the layer of cement around the pin. What happens? The tooth in the opposite jaw bites against the crown at this point, and there is a tendency to tip it backward. There is a constant strain against the cement, and moisture is permitted to enter, the cement is disintegrated, and off comes the crown.

As to the crowns put in with amalgam, I will say this: Logan originally began with a small round pin, and found that the amalgam eat up the pin. The pin that is used to-day in the Logan crown the amalgam does not eat up; but the tooth is not held as tightly as it can be with either oxyphosphate, cement or gutta-percha. In the case I spoke of, where the tooth came out last week, I do not suppose that its loss can be set down to the fault of anybody. The boy originally broke his tooth in a game of foot ball; then the crown which I put on was knocked off with a base-ball. He wore this last crown two years and a half, and then it was knocked off with a base-ball bat. The point which I wished to get at was to make a tool which would cut that canal the proper shape to receive the pin. This instrument does it. I sent the patterns to the manufacturer, and I take pleasure in saying that I was treated very considerately and handsomely. They took great pains to make the instruments as I wanted them, and after several changes were made they turned out the instruments as they are to-day, and which cut the canal very nicely indeed. The reamers are made in three sizes, and they make the hole to fit the pins.

The next point is in regard to the old method of setting those crowns. The crown comes shaped as we see it here (referring to drawing). The object was to be able to fit that to the natural root without tearing the gum. You had to use very small stones or chisels, and in nine cases out of ten you lacerate the gum to a considerable extent; and I think that is one of the causes of recession of the gums. I went to the dental depots to find a facer. Every facer I found was convex on the surface, so that it would cut a concave hole in the root, or they were simply burs; the result being that at the first turn of the instrument it would clog and would not cut any more. These instruments, which I have devised, are narrowed back from the point towards the shank, so that the cutting point is the widest point. Then these flanges allow the *debris* to run down between, so that the instrument will cut ahead *ad libitum.* When that instrument is put into the root of a tooth it cannot cut anything that is not already a canal. The further you go down the greater the difficulty of cutting, and the less the danger of going through the end or the side wall. From its rigidity it is impossible for it to turn a curve, as with some instruments. The instrument being curved, will take up the *debris*, which it cuts; and when you remove it from the tooth the *debris* in the groove will show exactly the depth to which you have cut; and that can be laid on the pin, and will show exactly how much room you have for the pin. Dip the instrument in a glass of water and it can be perfectly cleaned. The other instrument should also be cleansed after use, otherwise the blood and other material upon them will interfere with the cutting edge.

The way I set a crown is first to ream the canal so as to exactly fit the pin. But I want to warn you against being in a hurry, for one object I have is to depend not merely on the cement, or whatever material is used for setting the crown, but somewhat on the mechanical arrangement of the pin in the root. A cross section shows that the pin is flattened in the center, and has a flange on its two edges. If the reamer be allowed to cut down into the canal, and is simply moved backward and forward, it will drill an elipse, an elipse that will be perfectly rhomboidal; therefore, when the pin is put into the tooth it touches the walls of the canal at four points. There being a square surface and a grooved one, there is a space on both sides for cement. The depression in the tooth leaves room for the cement; consequently in addition to the holding power of the cement, you have that of the impingement of the pin upon the walls of the canal at four points.

I recently had a case where the crown of a central incisor was so badly decayed that I advised its removal and the setting of one of these crowns. The lateral also had a large cavity in it, and the patient wished to have the lateral cut off too; but I deemed it advisable to save it. I cut off the central and fitted my crown and pin, and was about to set it when I found the patient determined to have the other one cut off and crowned also. That I objected to, and referred the matter to her father. In the interim I filled the lateral with oxyphosphate and put a Logan crown on the central, not using any cement, for the reason that I wished to take it off subsequently, for the easier filling of the lateral. About that time I went to Albany, and the patient was out of the city for three months before I saw her again; then she came in determined to have the lateral cut off and crowned. I refused to do it and the result was that the case was left to me. She was going away again and wanted to know if the oxyphosphate filling could not be left until she come back. I was about to let her go away when it occurred to me that I had not fastened the Logan crown on. I tried to remove it, and found it impossible to do so with my fingers, To be sure about the matter I looked up the record, and found that the crown had not been set. But it was necessary to use the forceps to remove it. I did remove it and set it permanently. That circumstance is related simply to show the value of the mechanical adaptation of the pin to the root, irrespective of the cement. In order to get that perfect adaptation you must cut very slowly with the reamer, because while the reamer may be the exact size of the pin, it is only the exact size of the pin for a certain length, and not as far as the pin goes. If you send the reamer a little bit further, your canal will be too large for the pin. Therefore, I say cut a little and try your pin, then cut a little more and fit your pin again, and so on until you get it in, as you see it in the drawing here. The tooth must not, at first, go quite into place without the use of the mallet, and must be fitted so tightly that forceps will be required to remove it. That, I think, is the ideal way of fastening a crown in position.

A word of caution about the facers. They cut very rapidly provided the canal has been reamed large enough at the mouth to allow the tip to go down and rest on the tooth structure; and there is a great temptation to cut right down to the gum, which I have done to my cost. Be careful too, instead of cutting your tooth square, cut it at a slant, leaving as much of the tooth substance on the lingual or palatal aspect as there is in the mouth. I generally square it off, as you see, considerably above the gum line, then cut this side off and ream a little down into the cavity. I put in my pin, and if it does not touch at this point I ream a little deeper and try again. In nine cases in ten you will find that you will need to take very little off the palatal side.

If you have everything fitted properly, when you tap the tooth into position it will form a good joint. If the edges do not come out as smooth as they should they must be ground off with a smooth stone and disks in the mandril, used alternately. That edge must be squared up to fit against the square edges of the tooth. Perhaps it will not fit as well as you can get it with platinum burnished over the end of it, but well enough. And the crowns stay on.

I may say right here that I have not said much about grinding the crowns. I don't do that. If my tooth is too long for the bite I take it off the root. The pin comes quite near to the surface, and whatever you grind away for the bite weakens the tooth very materially. Every bit of material that you take away lessens the value of the crown if you take it away from around the pin.

In these cases where the roots are not straight, where the canal seems to be curved, if the teeth were to be set in accordance with the angle the point of the tooth would be out of line so that the articulation would interfere with the crown, and it then becomes necessary to bend the pin. It is very easily bent and then driven home in the same way as before. The bending of the pin almost invariably makes it necessary to have the joint above the gum line on the palatal side. Therefore first find out the direction of your root, and the direction of the pin can be changed to suit.

Now when you come to a case where the crown is not cut off, a case where the pulp has been dead for a long time and decay has gone on, there will be too large a hole for the use of the reamer. Suppose that the walls are still solid and the tooth sufficiently strong. It then makes a case for gutta-percha. The decay is removed as far as possible with spoon excavators, and the cavity scraped out, leaving the inside as rough as possible. In this chart you see the flanges which I spoke of on the pin. By cutting those flanges with a knife and bending the spurs down, it gives the pin a barbed appearance. The gutta-percha is placed around the pin and the pin driven into place, and it stays there.

In regard to gutta-percha swelling so as to split the root, I have only to say that you must use judgment in selecting the teeth in which to place it; you must not put gutta-percha in a tooth that is weak enough to be split by its expansion, a result which I think is not much to be feared. Now in cases that are set with amalgam, the space is first filled with amalgam, leaving the rough lines to hold the amalgam in, and while it is plastic an instrument is pressed into it and turned around in it, and the patient dismissed until the next day. By this method of restoring, with amalgam, the lost substance of the tooth, and reaming out of the amalgam a space for the pin, the root is considerably strengthened.

Here is a tooth that looks very much like a Logan crown, and I take great pleasure in bringing it to your attention to-night. I believe it has not been shown before. It was described to me in Boston by Dr. Blivan, of Worcester. I happened to ask him what crown he used and he described this. A piece of platinum is burnished over the end of the root. A hole is drilled through the center of it and the pin is fitted into the canal. He uses the Logan crown pin. He then takes a plate tooth with cross pins and grinds it, fitting it nicely and setting it at the desired angle, and allowing the two pins in the tooth to wrap around the pin that is set in the canal. He then packs tooth body around the pins and bakes it in the furnace. In this way he practically makes a Logan crown for each case. The only advantage is that he gets a perfect joint with the platinum.

Dr. Van Woert's crown is practically the same thing, with the exception that the final element is gold solder.

Another reason why I call your attention to this crown is that it will not only make a very pretty single crown, but can be made the attachment for bridge work without danger of infringing anybody's rights. After backing you can solder a bridge to it when that is desirable. My experience with the Logan crown tells me that this tooth, if properly and accurately set, will last as long as any other.

I don't wish to argue that any method is a failure because of the failure of single cases ; but it is remarkable that so many teeth roots have been lost that have been banded. I presume that is largely due to the way in which the bands are put on—because they are many times improperly fitted ; I have been obliged to abandon their use. I have seen Logan crowns fail, as in those that I put on before I made these reamers. It may be in some cases a band is a good thing, but I think that if there is any tendency to gingivitis, or similar diseases, a band is a very bad thing to put under the gum. And it seems to be not absolutely necessary, because the porcelain makes a prettier piece of work, and the joint is much better, and I have never seen a case where the gum has inflamed around a Logan crown.
bers of the society to defray the expense of publication. Dr. Levy offered an amendment that the treasurer make the assessment. Amendment accepted, and resolution adopted as amended.

*The President*—The discussion of Dr. Ottolengui's paper on the Logan crown, read last evening, is now in order.

*Dr. Thayer*—I would like to ask if Dr. Ottolengui has had any experience in the use of the Brown crown, and what his opinion is as to the comparative strength of that and the Logan crown.

*Dr. Ottolengui*—I did not say anything last night about the Brown crown, because I like Dr. Brown and I don't like his crown. There seems to me to be no advantage whatever in the straight or single pin tooth over the others, but in the double pin tooth I think there is a decided disadvantage. The double pin being set at two different points in the tooth, with a small portion of the substance of the tooth between the pins, that little portion of tooth substance between the pins is a point of weakness. It is the same with the Logan crown that has two pins. The pin is almost as tight as if it were vulcanized there, and when you tap the crowns into place they split. I have never broken a Logan crown until yesterday here. That was a double pin tooth, and it broke while being driven into position. I do not use the Brown crown for this reason. In the single pin I like a groove on the side of the Logan pin which gives a space for cement.

*Dr. Thayer*—Unlike Dr. Ottolengui, I think a great deal of Dr. Brown's crown, and, like the Doctor, I think a great deal of Dr. Brown. There is no question in my mind that there is a vast increase of strength in the Brown anterior tooth crowns. Dr. Brown constructs his crown in such a manner as to build up on the pin a small portion of the body which he bakes in the crown, and I think that adds a great deal of strength to the crown. The Logan crown is gouged out at this point (referring to diagram), and this is the point where the greatest strain comes upon an artificial crown, in my opinion, further down in the tooth; and that is why I consider the Brown crown vastly superior in strength to any other crown in the market, more than a hundred per cent. stronger.

*Dr. Littig*—I would like to ask Dr. Ottolengui how long he has been setting these crowns.

*Dr. Ottolengui*—I have been setting them ever since they were made. I think the new pin has been out about two and a half years.

*Dr. Littig*—Have been setting them in this way all that time?

*Dr. Ottolengui*—No. I think it is 18 or 19 months since I made the reamers. For that length of time I have been setting them in that way.

*Dr. Littig*—There have been many pivot crowns put on the market and a large proportion of them seem to have been failures. I don't think we have sufficient data to talk intelligently about them as yet. As to the Brown crown I cannot conceive how we could have a stronger crown simply by the addition of porcelain, because the strength is usually given to a tooth by pressure in the mold in the first place, and when we add to it we do not have the same degree of strength.

*Dr. Thayer*—The White Dental Manufacturing Company manufacture these teeth in a mold, and they have the pressure there. There is no question that if you get more body of porcelain there you have more strength, and do not weaken the root. If a little is strong a good deal is stronger; there is no question about that. They are both made in a mold.

The subject was passed.

[TO BE CONTINUED.]

